# Functional Leaf Traits and Diurnal Dynamics of Photosynthetic Parameters Predict the Behavior of Grapevine Varieties Towards Ozone

**DOI:** 10.1371/journal.pone.0135056

**Published:** 2015-08-13

**Authors:** Elisa Pellegrini, Alessandra Campanella, Marco Paolocci, Alice Trivellini, Clizia Gennai, Massimo Muganu, Cristina Nali, Giacomo Lorenzini

**Affiliations:** 1 Department of Agriculture, Food and Environment, University of Pisa, Via del Borghetto 80, 56124, Pisa, Italy; 2 Department of Agriculture, Forests, Nature and Energy, University of Tuscia, Via San Camillo de Lellis, 01100, Viterbo, Italy; University of Vigo, SPAIN

## Abstract

A comparative study on functional leaf treats and the diurnal dynamics of photosynthetic processes was conducted on 2-year-old potted plants of two grape (*Vitis vinifera* L.) varieties (Aleatico, ALE, and Trebbiano giallo, TRE), exposed under controlled conditions to realistic concentrations of the pollutant gas ozone (80 ppb for 5 h day^-1^, 8:00–13:00 h, + 40 ppb for 5 h day^-1^, 13:00–18:00 h). At constitutive levels, the morphological functional traits of TRE improved leaf resistance to gas exchange, suggesting that TRE is characterized by a potential high degree of tolerance to ozone. At the end of the treatment, both varieties showed typical visible injuries on fully expanded leaves and a marked alteration in the diurnal pattern of photosynthetic activity. This was mainly due to a decreased stomatal conductance (-27 and -29% in ALE and TRE, in terms of daily values in comparison to controls) and to a reduced mesophyllic functioning (+33 and +16% of the intercellular carbon dioxide concentration). Although the genotypic variability of grape regulates the response to oxidative stress, similar detoxification processes were activated, such as an increased content of total carotenoids (+64 and +30%, in ALE and TRE), enhanced efficiency of thermal energy dissipation within photosystem II (+32 and +20%) closely correlated with the increased de-epoxidation index (+26 and +22%) and variations in content of some osmolytes. In summary, we can conclude that: the daily photosynthetic performance of grapevine leaves was affected by a realistic exposure to ozone. In addition, the gas exchange and chlorophyll *a* fluorescence measurements revealed a different quali-quantitative response in the two varieties. The genotypic variability of *V*. *vinifera* and the functional leaf traits would seem to regulate the acclimatory response to oxidative stress and the degree of tolerance to ozone. Similar photoprotective mechanisms were activated in the two varieties, though to a different extent.

## Introduction

Ozone (O_3_) in the troposphere is the most damaging air pollutant affecting plant life and human health [1]. Despite air quality regulations intended to limit and/or mitigate O_3_ pollution, background mean concentrations in the Northern Hemisphere have more than doubled to 35–40 ppb since the industrial revolution [2] and in many regions daily peak levels continue to exceed the World Health Organization guideline values of 50 ppb [3]. In addition, O_3_ concentrations are expected to rise in developing countries due to increased emissions of nitrogen oxides (NO_x_) and other O_3_ precursors (such as hydrocarbons) resulting from anthropogenic activities.

This scenario threatens (i) local and global food security, (ii) fiber and timber production, (iii) conservation and genetic diversity of natural plant communities, and (iv) growth and yield of agricultural and horticultural plants [4]. In Mediterranean countries, where photochemical air pollution is high, ambient O_3_ episodes not only cause visible injury to crops and native plants, but also negatively affect plant performances [5–6].

Although the effects of O_3_ on annual crops have been widely analysed, few studies have investigated the sensitivity of perennial plants. To the best of our knowledge, fumigations of grapevines are scarce in spite of the fact that the grape had been the first plant where the first disease proved to be caused by O_3_ had been demonstrated [7]. For the first time in Europe, Lorenzini et al. [8] detected field O_3_ injury on several grape cultivars. Other observations in Spain and Greece confirmed the presence of O_3_-induced symptoms on this crop [9–10]. Some studies have been performed for a short exposure time with unrealistic high pollutant concentrations (e.g. [11]). In terms of leaf visible injury, grapevine is regarded as sensitive to O_3_ [8; 12, 13]. Damage caused by O_3_ on grapevine leaves includes structural changes in the cuticle and anatomical modifications at the mesophyll level inducing necrotic spots [14–15]. Since grapevine is a multi-annual crop, studies on its interaction with O_3_ should consider the possible long-term effects on carbohydrate metabolism, allocation of resources, and grape production. Acute O_3_ exposure has been found to damage the photosynthetic structures with increased chloroplast stroma electrondensity and modifications of thylakoid system [16–17]. Less information is available on the interaction of O_3_ on grape yield and quality. Soja et al. [18] showed that a 2-year exposure to O_3_ (8 h d^-1^, 5 d week^-1^) affected fruit yield and sugar concentrations in the juice because O_3_ impacted the photosynthetic capacity and carbohydrate metabolism.

Within a single species, differences in structural and functional traits may play important roles in adaptation/acclimation to environments characterized by high oxidative pressure. Functional traits can predict plant behavior in its natural environment [19] and have been correlated to the degree of tolerance to oxidative stress in forest trees [20]. Less information is available for *Vitis* in this context.

Differences in O_3_ sensitivity amongst species and daily trends must be taken into account in the prediction of O_3_ injury [21]. Considering the current scarce knowledge regarding O_3_ sensitivity within *V*. *vinifera* germplasm, which could include varieties cultivated in restricted areas with passive (constitutive; i.e. not responsive to current oxidant challenge) and/or active (inductive; i.e. responsive to current challenge) defense mechanisms against abiotic stress, the aim of this study was to assess the O_3_ stress-related responses of photosynthetic primary reactions. In addition we examined their relationships with net carbon assimilation, as well as the activation of photoprotective processes in two *V*. *vinifera* varieties with distinct vegetative vigor and berry characteristics.

We attempt to answer some key questions:

- Does O_3_ realistic exposures affect the daily photosynthetic performance of grapevines leaves?
- Can the grapevine’s genotypic variability and functional leaf traits (*sensu* Bussotti and Pollastrini [22]) regulate acclimation to oxidative stress?
- What about the role(s) played by the mechanisms that underlie xanthophyll cycle-dependent thermal energy dissipation, photorespiration and stomatal closure?

## Materials and Methods

### Plant material and ozone exposure

Preliminary results of previous O_3_ fumigation carried out on six *V*. *vinifera* varieties widely grown in central Italy enabled us to select the black-berried cv. Aleatico (ALE) and the white-berried cv. Trebbiano giallo (TRE) for this experiment. These two varieties showed a strong differential response to oxidative stress in terms of the modulation of non-enzymatic antioxidant systems (such as ascorbic acid, Pellegrini et al. unpublished data). Studies aimed at analyzing responses to biotic stresses showed that TRE has a higher resistance to downy mildew (*Plasmopara viticola*) compared to ALE: disease incidence was significantly lower in TRE because of the lower number of infected stomata per leaf surface unit [23].

Potted plants of both varieties were obtained by woody cuttings derived from field-grown donor plants and rooted in 5 l pots containing a 2:1 mixture of commercial peat and pumice. No specific permissions were required for this plant material because the owners of the land and of the field-grown donor plants are the University of Pisa and University of Tuscia (Italy), respectively.

Experimental activities were conducted in the field-station of San Piero a Grado, Pisa (43°40′N, 10°21′E), Tuscany, Italy. Before plant budbreak, homogeneous two-years-old plants were fertilized with Osmocote NPK 10-11-18. The experiment was performed into a greenhouse fumigation facility, in a total of four closed Perspex boxes (measuring 0.90 × 0.90 × 0.65 m). Two boxes were continuously ventilated with charcoal filtered air (two complete air changes/min) and used as controls. The other two boxes were ventilated with O_3_-enriched air. The 28^th^ of April, uniformly sized plants were placed into the boxes under controlled irrigation for one month and in filtered air (O_3_ concentration was negligible, below 5 ppb, as measured by a photometric O_3_ analyzer, Monitor Labs, mod. 8810, San Diego, CA, USA). The 28^th^ of May, plants of each variety chosen at random were splitted in two sets and catalogued as control and O_3_-treated set. The plants catalogued as control set were placed into two boxes continuously ventilated with charcoal filtered air (each box housed both varieties). Plants catalogued as O_3_-treated set were placed into two boxes ventilated with O_3_-enriched air (each box housed both varieties). O_3_ was generated by electrical discharge using a Fisher 500 air-cooled apparatus (Zurich, Switzerland) supplied with pure oxygen, and mixed with the inlet air entering the fumigation chambers. O_3_ concentration at plant height was continuously monitored with a photometric analyzer connected to a computer [24].

The exposure conditions in each chamber were the same during the whole experiment (plant growth and O_3_-exposure). The minimum and maximum temperature and relative humidity (RH) of air were 16–27°C and 60–55%, respectively. These values were recorded by four Tinytag Ultra 2 Data Loggers (Gemini Data Loggers, Chichester, UK) (each box housed one Data Logger). Average daily solar radiation was around 250 W h m^-2^ at plant height (LI-190R Quantum Sensor, LI-COR, Nebraska, USA).

Fumigations were carried out before plant flowering, from 28 May to 24 June 2012 (28 days) with a pulse of O_3_ concentration between 08:00 and 13:00 daily (daylight saving time, DST), with a target concentration of 80 ppb (for O_3_, 1 ppb = 1.96 μg m^-3^, at 20°C and 101.325 kPa) and, between 13:00 and 18:00 (DST) daily, with 40 ppb. The fumigation simulated the typical summer circadian profile of O_3_ in central Italy, as calculated by historical data (1994–2010) for the photometric analyzers network in Tuscany.

Leaf photosynthetic carbon dioxide (CO_2_) assimilation (A) responses to irradiance and to intercellular CO_2_ concentration (C_i_) were analyzed on fully expanded leaves at 7, 14, 21 and 28 days from the beginning of exposure (FBE). At the end (28 days), circadian analyses (gas exchange, modulated chlorophyll *a* fluorescence) were carried out every two hours, beginning at dawn (06:00–20:00 h DST). Leaf water status (predawn, Ψ_PD_, midday,Ψ_2_, water and predawn osmotic, Ψ_osm_, potential) and the relative water content (RWC) were determined. For each sampling time, leaf samples were collected in order to determine metabolite levels: abscisic acid (ABA), proline, photosynthetic pigments and water soluble carbohydrates (WSC). Optical and electron microscopy observations (quantification of stomata number, foliar anatomy and leaf senescence) were conducted in order to characterize (i) the constitutive leaf functional traits of both varieties and (ii) the effects of O_3_ treatment.

### Ecophysiological measurements

CO_2_ and water vapor exchanges were measured with an open infra-red gas exchange system (CIRAS-2, PP-Systems, Amesbury, Massachusetts, USA) equipped with a Parkinson leaf chamber for clamping single leaves. Measurements were performed at ambient CO_2_ concentrations (340–360 ppm) at 80% RH. The chamber was illuminated by a quartz halogen lamp, and leaf temperature was maintained at 26±0.4°C. Photosynthetic activity was measured at 1200 μmol photons m^-2^ s^-1^. The calculation of C_i_ was based on the equations described by von Caemmerer and Farquhar [25].

Measurements were taken from six plants and repeated to obtain at least six stable readings for each marked leaf. Leaf photosynthetic CO_2_ assimilation responses to irradiance were calculated using Smith’s equation [26], determined at a specific CO_2_ concentration of 340 ppm. Photosynthetic photon flux density (PPFD) was decreased from 1600 to 0 μmol photon m^-2^ s^-1^ in 15 steps (1600, 1400, 1200, 1000, 800, 600, 400, 300, 200, 150, 100, 80, 40, 20 and 0 μmol photon m^-2^ s^-1^).

Photosynthetic activity was recorded after stabilization at each light intensity. Three measurements were recorded at 2-min intervals for each PPFD level per leaf. The first recording was performed after the leaf had adjusted to the highest light intensity (PPFD = 1600 μmol photon m^-2^ s^-1^) for 5 min in order to obtain a maximal stomatal aperture [27]. The Light Compensation Point (LCP), the Light Saturation Point (LSP) and photosynthetic activity at saturating light level (A_max_) were calculated according to Surabhi et al. [28]. The relationship between A and C_i_ (C_i_ < 200 ppm) was analyzed according to Sharkey’s mechanistic model of CO_2_ assimilation [29]. The CO_2_ concentration was increased from 0 to 1800 ppm (0, 20, 40, 80, 100, 150, 200, 400, 600, 800, 1000, 1200, 1400 and 1800 ppm). The maximum carboxylation rate of Rubisco (V_cmax_) and the light-saturated rate of electron transport (J_max_) were calculated according to Dubois et al. [30]. The assimilation chamber was maintained at 63±7.0% RH and a temperature of 25±1.1°C.

Modulated chlorophyll *a* fluorescence and the status of the electron transport of photosystem II (PSII) were measured at room temperature with a PAM-2000 fluorometer (Walz, Effeltrich, Germany) on the same leaves used for gas exchanges dark-acclimated for 40 min, using a dark leaf clip. To determine the minimal fluorescence level (F_0_), where all primary quinone acceptors of PSII are oxidized and capable of photoreduction, the leaf was exposed to a very weak, modulated measuring beam, which was sufficiently low to prevent any significant variable fluorescence. The maximal fluorescence level (F_m_), when all PSII centers were closed, was determined by applying a saturating light pulse (0.8 s). The saturating pulse method was also used to analyse photochemical quenching (qP) and non-photochemical quenching (qNP), as described by Schreiber et al. [31]. Fifty intermittent pulses of saturating strong white light (0.8 s at 15,000 μmol m^-2^ s^-1^) were applied every 20 s in the presence of actinic light (about 400 μmol m^-2^ s^-1^). After the saturating pulse, the actinic light allowed to reach steady-state photosynthesis and to determine the maximal fluorescence in the light-adapted state (F’_m_) and the fluorescence yield at this steady-state (F_s_).

After removing the actinic light source, the minimal fluorescence level in the light-adapted state (F’_0_) was immediately determined in the presence of a far-red (> 710 nm) background for 10 s to ensure maximal oxidation of PSII electron acceptors. Φ_PSII_ was calculated as (F’_m_—F_s_) / F’_m_, where F_s_ is the steady-state fluorescence yield in the light-adapted state, as described in Rohacek [32]. The fraction of absorbed light that was thermally dissipated in the PSII antennae (%D) was estimated from 1 − (F’_v_ / F’_m_) × 100, according to Demmig-Adams et al. [33].

The plant water status was determined on whole leaves using a sealed pressure chamber (model 600 Pressure Chamber Instrument, PMS Instrument Company, Albany, NY, USA) and nitrogen (N_2_) for the application of pressure. The individual plant moisture stress (PMS) of the leaf was defined as soon as the first drop appeared on the petiole. Each leaf was measured and three replications were taken at 6:00 and at 14:00 h. To determine the osmotic leaf water potential (Ψ_osm_), 500 mg of frozen plant material were thawed for 30 s, major veins were excised and 10 μl of sap were squeezed out for the determination of the solute concentration [34] with a Wescor 5500 Vapor Pressure Osmometer (Livingston, UK). RWC was determined according to Nali et al. [24] and expressed as 100 × (FW—DW) / (SW—DW), where FW, DW and SW were fresh, dry and saturated weights, respectively.

### Biochemical measurements

ABA was determined according to Pellegrini et al. [35] by an indirect enzyme-linked immunosorbent assay (ELISA), based on the use of a monoclonal antibody raised against S(+)-ABA. Leaf samples (100 mg FW) were collected, weighed and stored at -80°C until the analysis. ABA was measured after extraction in distilled water (water:tissue ratio = 10:1, v/w) overnight at 4°C. Plates were coated with 200 μl per well ABA-4’-BSA conjugate and incubated overnight at 4°C, then washed three times with a 75 mM PBS buffer, pH 7.0, containing 1 g l^-1^ BSA and 1 ml l^-1^ Tween 20, keeping the third washing solution for 30 min at 37°C. In the next step, 100 μl ABA standard solution or sample and, subsequently, 100 μl DBPA1 solution (lyophilized cell culture medium diluted in PBS buffer containing 10 g l^-1^ BSA and 0.5 ml l^-1^ Tween 20, at a final concentration of 50 μg ml^-1^) were added to each well. Competition was allowed to take place at 37°C for 30 min. The plates were then washed again as described above, and 200 μl per well of a secondary antibody (alkaline phosphatase-conjugated rabbit anti-mouse—Sigma, Milan, Italy—in a PBS buffer containing 10 g l^-1^ BSA and 0.5 ml l^-1^ Tween 20, at a final dilution of 1:2,000) was added and incubated for 30 min at 37°C. The plates were washed again, 200 μl per well of p-nitrophenyl phosphate was added and then incubated for 30 min at 37°C. Absorbance readings at 415 nm were obtained using a microplate reader (MDL 680, Perkin-Elmer, Waltham, MA, USA).

Proline content was determined according to Bates et al. [36]. Plant material (100 mg FW) was ground in an ice-cold mortar with 2 ml of 3% sulfosalicylic acid. The homogenates were centrifuged for 30 min at 10,000 *g* at 4°C. The supernatant was filtered through 0.2 μm Minisart SRT 15 aseptic filters, and 1 ml of the filtrate was mixed with equal volumes of glacial acetic acid and ninhydrin reagent (1.25 g ninhydrin, 30 ml of glacial acetic acid, 20 ml 6 M H_3_PO_4_) and incubated for 1 h at 100°C. The reaction was stopped by placing the test tubes in ice-cold water. The samples were rigorously mixed with 2 ml toluene. After 20 min, the light absorption of the toluene phase was estimated at 520 nm, using toluene as a blank. The proline concentration was determined using a standard curve and calculated on a FW basis.

For carbohydrate analyses [37], the leaves (60 mg FW) were ground and homogenized in 1 ml of demineralized water for HPLC and heated for 60 min in a water bath at 60°C. The samples were then centrifuged for 20 min at 5,000 *g* at 20°C. Glucose, fructose and sucrose were determined from the supernatant. Carbohydrates were determined by injection of 20 μl sample volume into an HPLC system using a refill separation column (Sugar SC1018, 8 mm internal diameter × 300 mm length). Column temperature was 70°C and distilled water for HPLC was used as mobile phase (flow rate 0.8 ml min^-1^). Carbohydrates were detected with a differential refractometer (Shodex, West Berlin, NJ, USA) and pure authentic standards were used to quantify the carbohydrate content of each sample. The sum of glucose, fructose and sucrose was considered as a measure of WSC.

Photosynthetic pigments were determined by HPLC according to Döring et al. [38]. Thirty mg of leaves were dark-adapted for 30 min and homogenized in 3 ml 100% HPLC-grade methanol and incubated overnight at 4°C in the dark. The supernatant was filtered through 0.2 μm Minisart SRT 15 aseptic filters and immediately analyzed at room temperature with a reverse-phase Dionex column (Acclaim 120, C18, 5 μm particle size, 4.6 mm internal diameter × 150 mm length). The pigments were eluted at a flow rate of 1 ml min^-1^ using 100% solvent A (acetonitrile/methanol, 75/25, v/v) for the first 14 min to determine all xanthophylls, together with the separation of lutein from zeaxanthin, followed by a 3 min linear gradient to 100% solvent B (methanol/ethylacetate, 68/32, v/v), 15 min with 100% solvent B for the elution of chlorophyll *b*, *a* and β-carotene. The pigments were detected at 445 nm. Authentic standards were used to quantify the pigment content of each sample.

### Histological sampling, optical and electron microscopy

At the end of the treatment, three blade sections (2x2 mm, avoiding the major veins) per each leaf detached during the light phase were taken by a razor blade and fixed for 1 h at 4°C with a mixture of 4% paraformaldehyde and 5% glutaraldehyde in 0.1 M sodium cacodylate at pH 7.2, rinsed overnight in the same buffer, post-fixed in 1% OsO_4_ for 1 h at 4°C, and dehydrated in a graded ethanol series (50 to 100%).

A scanning electron microscope (SEM) (JSM 6010 LA, Jeol, Tokyo, Japan) was used to quantify the number of stomata and to characterize the abaxial leaf surface. The dehydrated samples were critical-point dried in a K850 apparatus (EMI-Tech, Timpson, TX, USA) equipped with a liquid CO_2_ inlet and metal shadowed in a gold sputtering unit equipped with an argon inlet (MED 010, Balzers Union, Liechtenstein). Specimens were then examined by SEM.

The dehydrated samples were infiltrated with a graded mixture of ethanol (2:1 for 1 h at 4°C; 1:1 for 2 h at 4°C; 1:2 for 2 h at 4°C), then embedded in Spurr resin polymerized at 70°C for 8 h before semi-thin sections (1 μm) and ultra-thin sections (70 nm) were cut on an Ultracut ultramicrotome (Reichert-Jung, Wetzlar, Germany). Semi-thin sections stained with blue toluidine were examined with the Axiophot optical microscope (Carl Zeiss, Oberkochen, Germany), and digital images for each sample were analyzed with the Axiovision 4 software (Carl Zeiss).

Foliar anatomy and cell ultrastructure were assessed by a transmission electron microscope (TEM). Ultra-thin sections were collected on copper grids, stained with uranyl acetate and lead citrate, and examined with a Jeol 1200 EX II TEM.

### Statistical analysis

After a Shapiro-Wilk W test had been carried out, data were analyzed using repeated measures (in the case of the measurements carried out for more than two time-points) or two-way analysis of variance (ANOVA) and comparison among means was determined by least significant (LSD) Fisher’s multiple comparison test (*P*≤0.05). Data of the (i) constitutive functional traits, (ii) leaf characteristics, and (iii) leaf water status characteristics were analyzed by Student’s *t*-test. All analyses were performed by NCSS 2000 Statistical Analysis System software.

## Results

**Fig 1:**
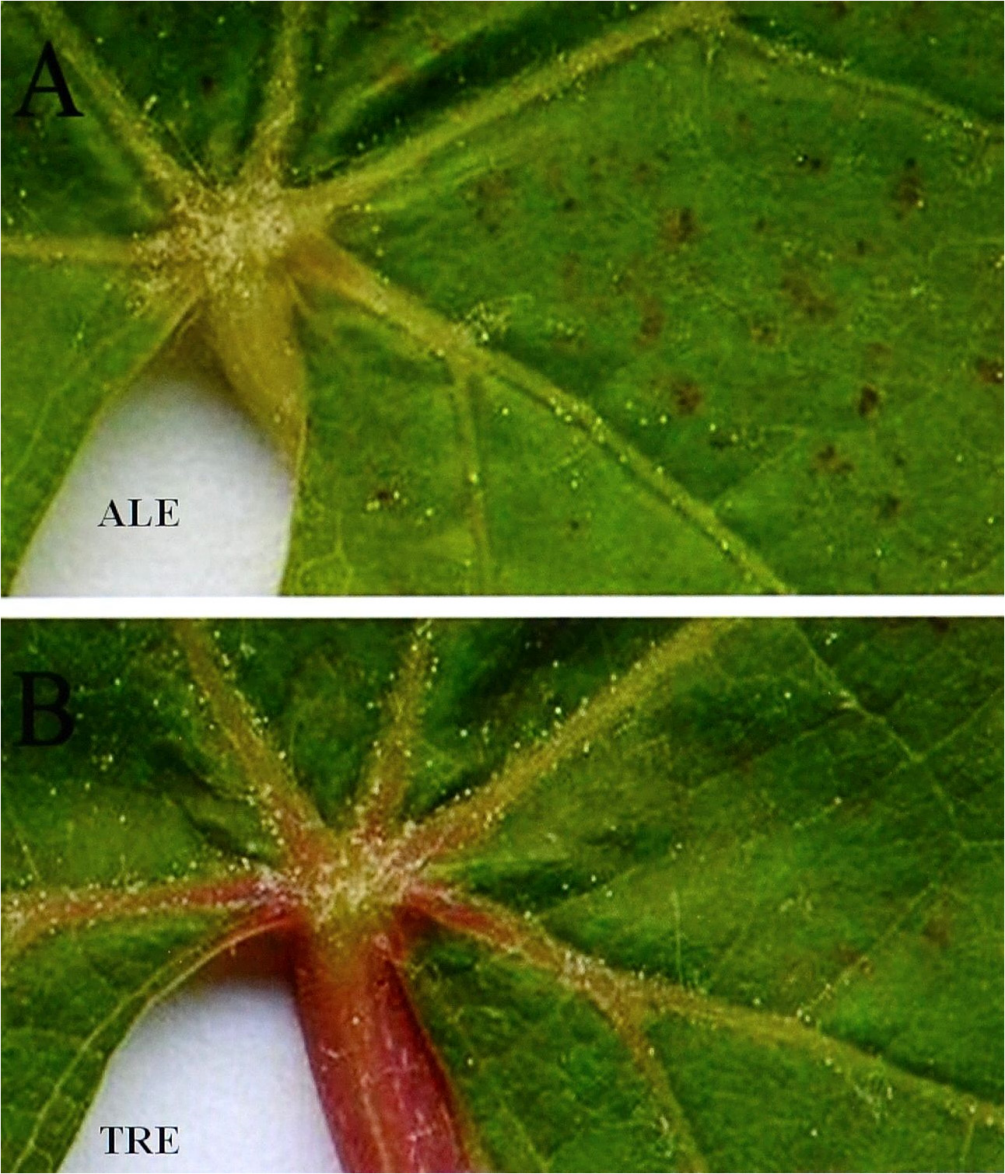
Macroscopic response of *Vitis vinifera* varieties to ozone treatment. Interveinal visible spots of browning tissue of *Vitis vinifera* leaves (Aleatico, A, and Trebbiano giallo, B) from plants fumigated with 80 ppb of O_3_ for 5 h day^-1^ (8:00–13:00 h) + 40 ppb of O_3_ for 5 h day^-1^ (13:00–18:00 h) for 28 days.

At the time of the leaf sampling of fumigated plants, O_3_-treated mature leaves of both varieties showed visible stipple (punctate spots, diameter about 1 mm) of browning tissue between veins of the adaxial surfaces (Fig 1). No damage was observed in unfumigated (control) plants.

### Optical and electron microscopy observations

Characterization of leaf constitutive functional traits (Table 1) showed a higher average value of the whole leaf thickness (LT) and of the leaf mass per area (LMA) in TRE compared with ALE (+13% for both parameters). Leaf longevity was lower in ALE, which showed earlier leaf drop compared to TRE. In addition, a lower number of stomata per surface unit was observed in TRE (-50%, *P*≤0.001, Student’s *t*-test) (Table 2). Palisade thickness was significantly affected by O_3_ (-15%) in ALE, but not in TRE (Table 2).

**Table 1:** Leaf constitutive functional traits in *Vitis vinifera* varieties.

Grape variety	Leaf thickness (μm)	Leaf mass area (mg cm^-2^)	Leaf longevity
**Aleatico**	139.2 ±2.3	6.9 ±0.8	Low
**Trebbiano giallo**	157.0 ±3.5	7.8 ±0.6	High
***P***	*	*	

Average values (± standard deviation) of leaf constitutive functional traits measured on two *Vitis vinifera* varieties (Aleatico and Trebbiano giallo) maintained in filtered air. For each parameter, the data were analyzed by Student’s *t*-test. The significant differences are: * = P≤0.05.

**Table 2:** Leaf characteristics in *Vitis vinifera* varieties exposed or not to ozone treatment.

Treatment	Stomata density (No. mm^-2^) (abaxial surface)	Palisade thickness (μm)
**Aleatico control**	277±28.8	52±4.2
**Aleatico fumigated**	262±46.4	44±2.1
***P***	ns	***
**Trebbiano giallo control**	138±23.8	56±3.4
**Trebbiano giallo fumigated**	115±19.6	48±2.8
***P***	ns	ns

Average values (± standard deviation) of leaf characteristics measured on two *Vitis vinifera* varieties (Aleatico and Trebbiano giallo) fumigated with O_3_ (80 ppb for 5 h day^-1^, 8:00–13:00 h + 40 ppb for 5 h day^-1^, 13:00–18:00 h) or maintained in filtered air for 28 days. Measurements referred to the end of the exposure. For each parameter, the data were analyzed by Student’s *t*-test. The significant differences are: *** = *P*≤0.001, ns = *P*>0.05.

**Fig 2:**
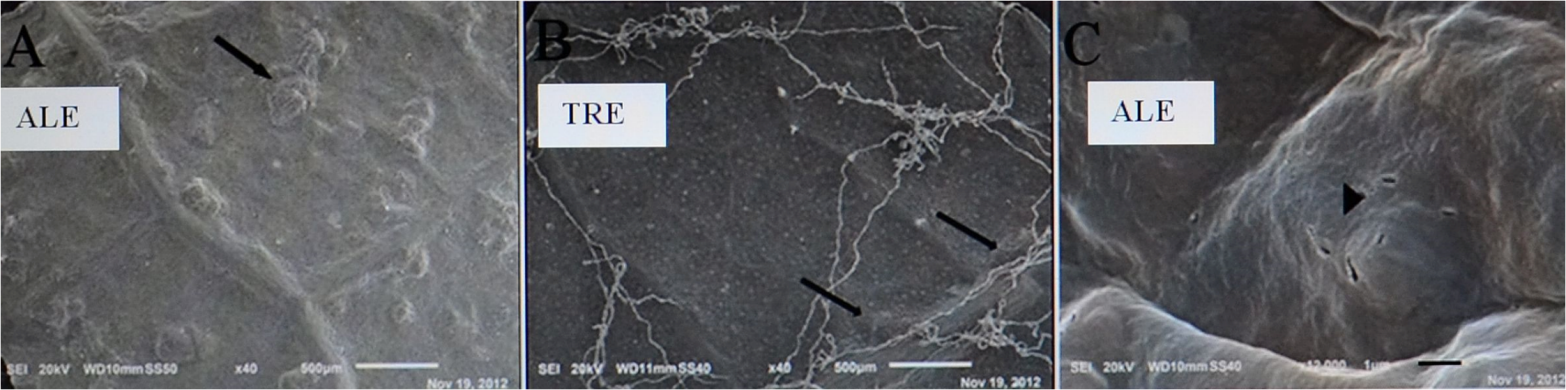
Microscopic response of *Vitis vinifera* varieties to ozone treatment observed by SEM. Micrographs of abaxial leaf surfaces observed by SEM in *Vitis vinifera*: Aleatico (A) and Trebbiano giallo (B) treated with 80 ppb of O_3_ for 5 h day^-1^ (8:00–13:00 h) + 40 ppb of O_3_ for 5 h day^-1^ (13:00–18:00 h) for 28 days. Arrows show deformed swelling areas. The magnification of these areas in Aleatico (C) revealed skin cracks on the epidermis (arrowhead). Bar marker is 500 μm (A-B) and 1 μm (C).

**Fig 3:**
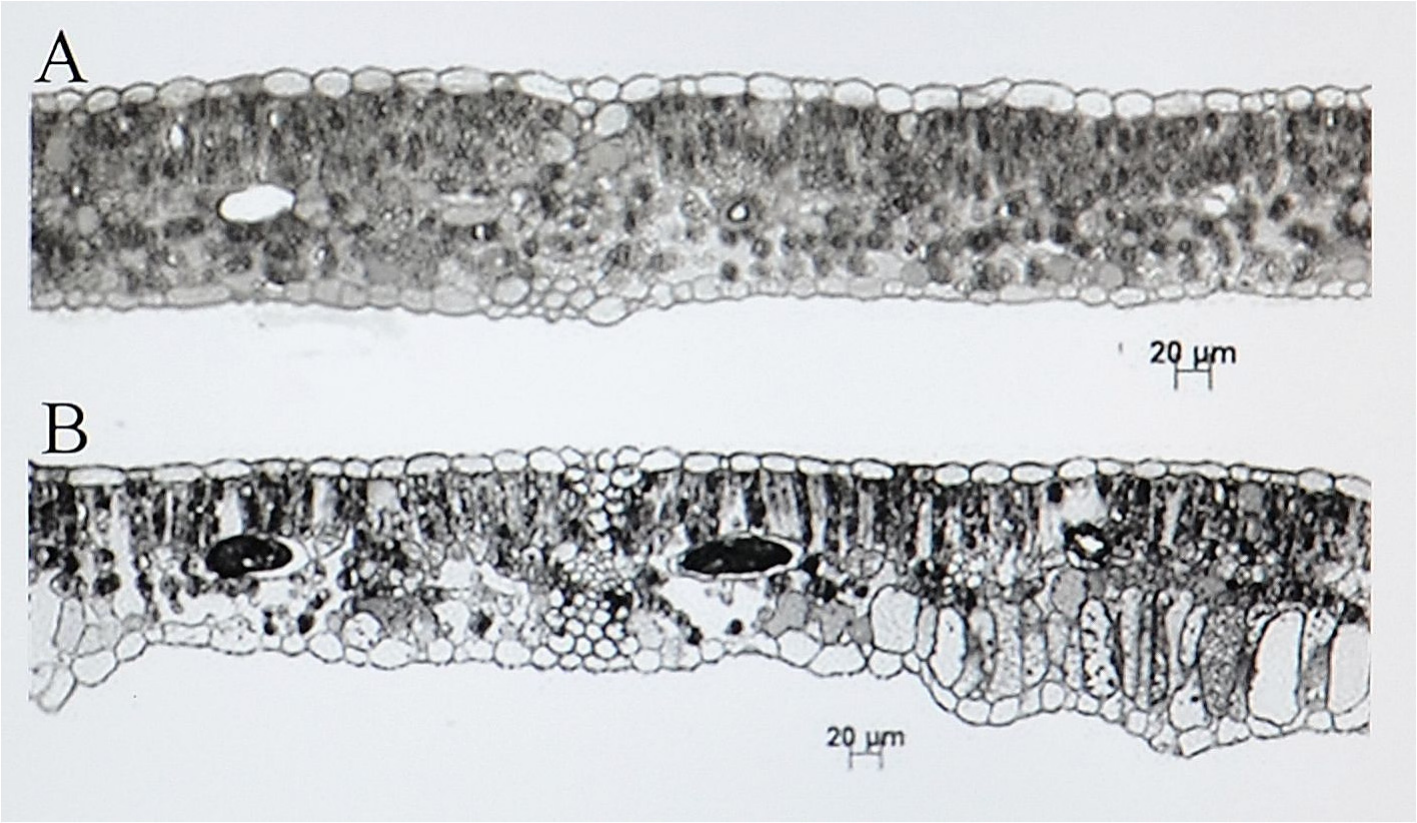
Microscopic response of *Vitis vinifera* cv. Aleatico to ozone treatment observed by light microscopy. Leaf semi-thin cross sections in *Vitis vinifera* cv. Aleatico (10 μm thick) observed by light microscopy. Plants were maintained in filtered air (A) or treated with 80 ppb of O_3_ for 5 h day^-1^ (8:00–13:00 h) + 40 ppb of O_3_ for 5 h day^-1^ (13:00–18:00 h) for 28 consecutive days (B): modifications of mesophyll anatomy and irregular increase in leaf thickness.

**Fig 4:**
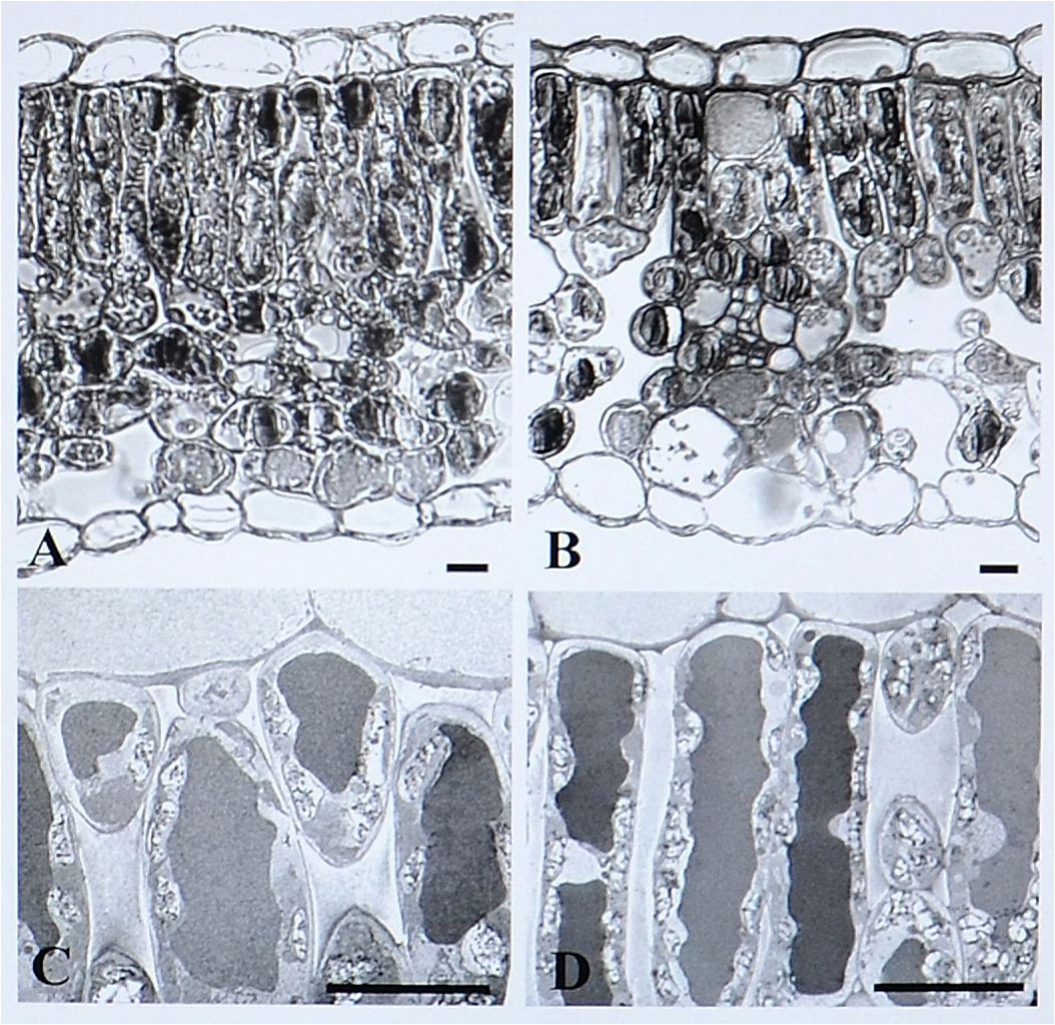
Microscopic response of *Vitis vinifera* cv. Aleatico to ozone treatment observed by light and transmission electron microscopy. Micrographs of ultra-thin sections observed by light microscopy (70 nm thick; A-B) and details of palisade layer observed by transmission electron microscopy in *Vitis vinifera* cv. Aleatico. Plants were maintained in filtered air (A; C) or treated with 80 ppb of O_3_ for 5 h day^-1^ (8:00–13:00 h) + 40 ppb of O_3_ for 5 h day^-1^ (13:00–18:00 h) for 28 days (B; D).Bar marker is 10 μm (A-B), 20 μm (C) and 30 μm (D).

SEM observations of the abaxial leaf surface of treated samples showed irregular deformed swelling areas among veins (Fig 2A and 2B). In ALE, magnification of these areas revealed skin cracks on the epidermis (Fig 2C). Modifications of the anatomy of ALE fumigated leaves were highlighted by leaf semi-thin cross sections, which showed an irregular leaf thickness (Fig 3) and modification of the palisade layer (Fig 4A and 4B). TEM observations revealed more detailed information on the reduction in cell contact in the palisade layer of treated ALE (Fig 4C and 4D).

**Fig 5:**
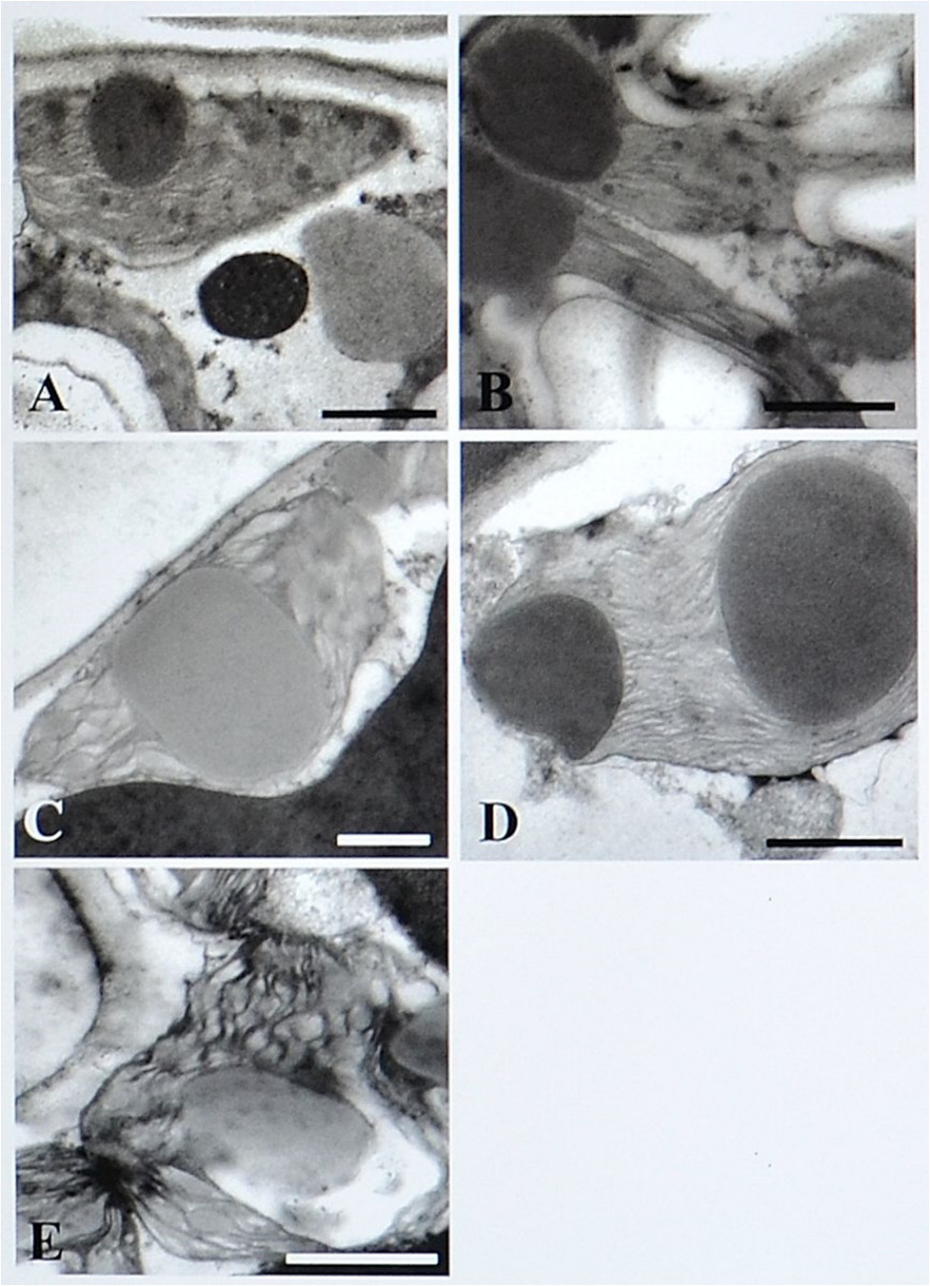
Microscopic response of *Vitis vinifera* varieties to ozone treatment observed by transmission electron microscopy. Micrographs of palisade cell chloroplasts in *Vitis vinifera* plants observed by transmission electron microscopy. (A) Aleatico and (B) Trebbiano giallo controls: presence of elongated chloroplasts and regular thylakoid system. (C) Aleatico and (D) Trebbiano giallo treated with 80 ppb of O_3_ for 5 h day^-1^ (8:00–13:00 h) and 40 ppb of O_3_ for 5 h day^-1^ (13:00–18:00 h) for 28 days: increase in the size of the plastoglobuli. (E) Aleatico treated: degradation of thylakoid system. Bars represent 1 μm.

Fig 5 shows the effects of oxidative stress on cell ultrastructure, particularly on the chloroplast characteristics: cross-sections of controls of both varieties showed elongated chloroplasts with a regular thylakoid system and a variable number of plastoglobuli (Fig 5A and 5B). An increase in plastoglobuli size was observed in treated material of both varieties (Fig 5C and 5D). Evidence of an increased degradation of the thylakoid system was observed in a large number of chloroplasts in treated ALE (Fig 5E).

### Dynamics of gas exchange

**Fig 6:**
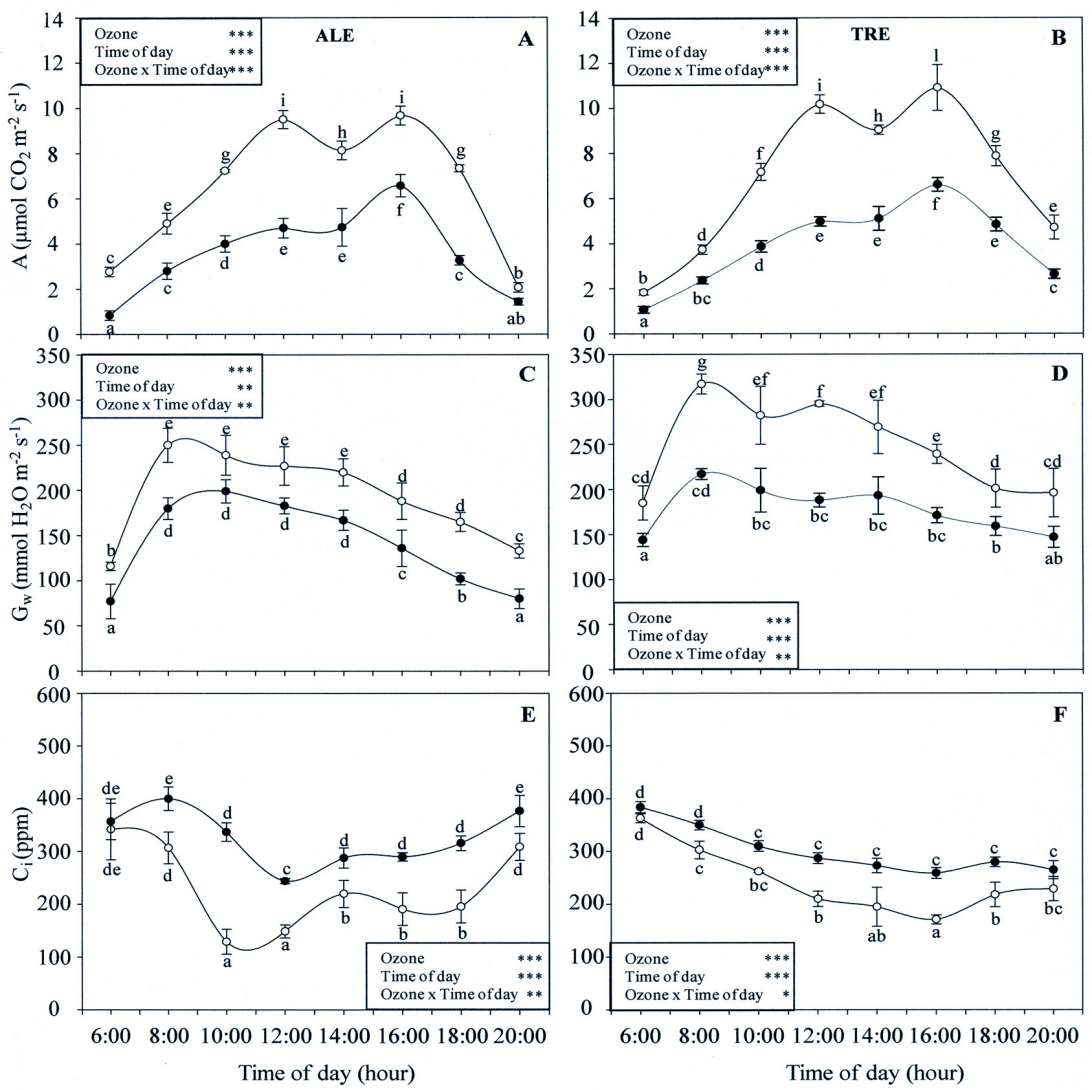
Diurnal time courses of gas exchange parameters in *Vitis vinifera* varieties exposed or not to ozone treatment. Diurnal time courses of assimilation rate (A, A-B), stomatal conductance to water vapour (G_w_, C-D) and intercellular CO_2_ concentration (C_i_, E-F) in *Vitis vinifera* leaves (Aleatico, left, and Trebbiano giallo, right) after a fumigation with 80 ppb of O_3_ for 5 h day^-1^ (8:00–13:00 h) and 40 ppb of O_3_ for 5 h day^-1^ (13:00–18:00 h) for 28 days. Data are shown as mean ± standard deviation. The measurements were taken from untreated (open circle) and treated (closed circle) plants at the end of exposure. For each parameter, different letters indicate significant differences (*P*≤0.05). In the boxes, results of repeated measurements ANOVA are reported. Asterisks show the significance of factors (ozone and time of day) and their interaction for: *** = *P*≤0.001, ** = *P*≤0.01, * = *P*≤0.05.

The circadian time course of gas exchange parameters is reported in Fig 6. The repeated measures of the ANOVA test revealed that the interaction between O_3_ and time of day, as well as the effects of both factors, were significant for all parameters and for both varieties. The A profile showed a biphasic diurnal pattern in ALE control plants (Fig 6A). The values were low early in the morning, increasing with time, and reaching a maximum at 12:00 and at 16:00 h. Afterwards, the levels gradually decreased, reaching a minimum at 20:00 h. Oxidative stress induced a strong quantitative change in the circadian profile of A: values were significantly reduced throughout the day in comparison with the control (except at 20:00 h), and a double-peak curve was obtained at 12:00 h (less marked in comparison to control, 50%) and at 16:00 h (-32%). Also in TRE controls (Fig 6B), the diurnal behaviour curve of A appeared to be a marked double-peak: the first peak appeared at 12:00 h and the second one at 16:00 h. O_3_ affected the A profile of TRE in the same way as ALE: a double-peak was observed at 12:00 h (-51%) and at 16:00 h (-44% in comparison with the control). Stomatal conductance to water vapour (G_w_) in untreated ALE exhibited peak values between 8:00 and 14:00 h (Fig 6C), followed by a gradual decline in the afternoon. Ozone did not affect the qualitative profile of G_w_, but modified its amount (e.g. -28% in treated leaves at 8:00 h). These findings were true not only for TRE controls (Fig 6D). In fact, O_3_ also induced the same reduction (e.g. -31% in treated leaves at 8:00 h). In both varieties, C_i_ was high in early morning (Fig 6E and 6F) and gradually decreased, reaching a minimum at 10:00 in ALE and at 16:00 h in TRE. Under oxidative stress, values were significantly higher, with the exception of those measured at 6:00 h in both ALE and TRE and at 20:00 h in TRE.

**Fig 7:**
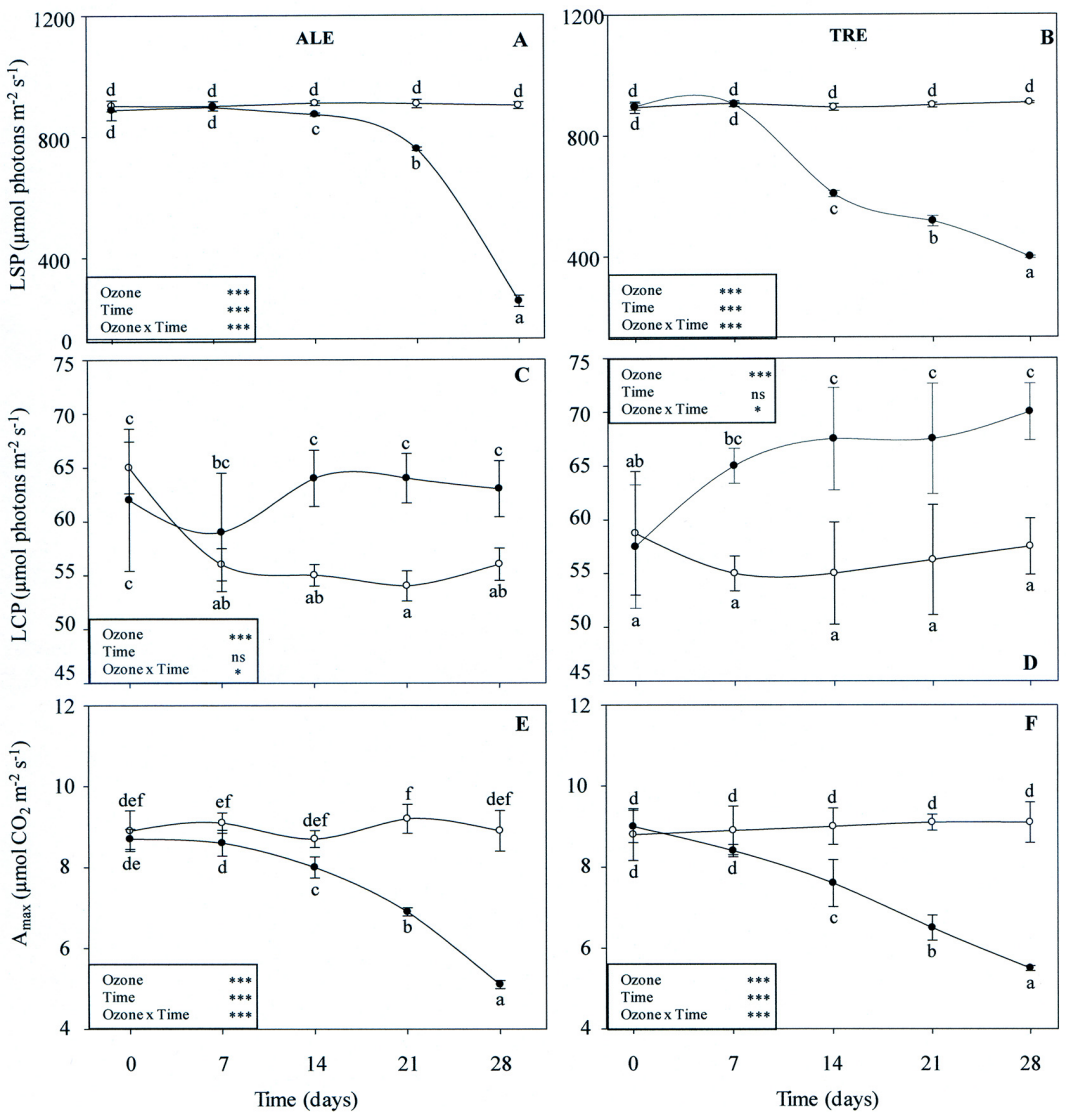
Weekly profiles of light curve-parameters derived in *Vitis vinifera* varieties exposed or not to ozone treatment. Weekly profiles of light saturation point (LSP, A-B), light compensation point (LCP, C-D) and net photosynthetic rate at light saturation point (A_max_, E-F) in *Vitis vinifera* leaves (Aleatico, left, and Trebbiano giallo, right) after a fumigation with 80 ppb of O_3_ for 5 h day^-1^ (8:00–13:00 h) and 40 ppb of O_3_ for 5 h day^-1^ (13:00–18:00 h) for 28 days. Data are shown as mean ± standard deviation. The measurements were taken from untreated (open circle) and treated (closed circle) plants. For each parameter, different letters indicate significant differences (*P*≤0.05). In the boxes, results of repeated measurements ANOVA are reported. Asterisks show the significance of factors (ozone and time of day) and their interaction for: *** = *P*≤0.001, * = *P*≤0.05, ns = *P*>0.05).

Irradiance response curves of the CO_2_ assimilation rate were measured in leaves exposed to filtered air or to O_3_ after 7, 14, 21 and 28 days of treatment (Fig 7). Interactions between O_3_ and time were always significant. The same was true for the effects of both single factors, with the exception of time for LCP in both varieties. In treated ALE, a slight reduction in LSP was observed starting from 14 days FBE (-3% compared with air filtered material). This decrease reached a maximum of -52% at the end of the treatment (Fig 7A). In treated TRE, the reduction at 14 days FBE was more pronounced (-22%), reaching the maximum at the end of fumigation (-39%) (Fig 7B). In both varieties, O_3_ induced an increase in LCP starting from 14 FBE in ALE and already at 7 FBE in TRE (+16 and +22%, respectively) (Fig 7C and 7D). This trend lasted until the end of the treatment. A reduction in the rate of A_max_ of fumigated leaves was observed after 7 and 14 days FBE in ALE (-5%) and TRE (-15%), respectively (Fig 7E and 7F). The highest decrease occurred at the end of the exposure (-43% in ALE and -40% in TRE).

**Fig 8:**
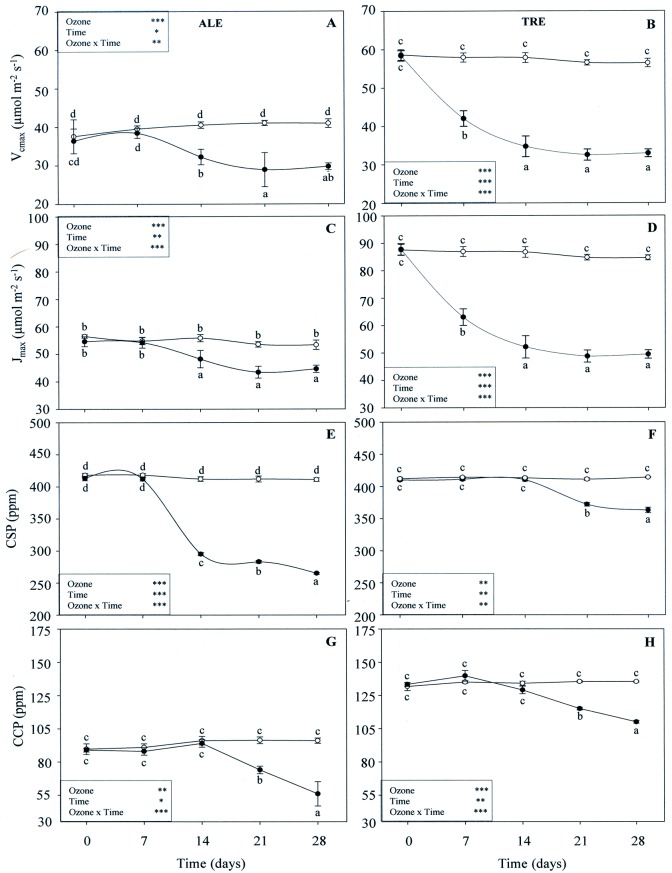
Weekly profiles of A/C_i_ curve-parameters in *Vitis vinifera* varieties exposed or not to ozone treatment. Profiles of maximum rate of Rubisco activity (V_cmax_, A-B), light-saturated rate of electron transport (J_max_, C-D), CO_2_ saturation point (CSP, E-F) and CO_2_ compensation point (CCP, G-H) in *Vitis vinifera* leaves (Aleatico, left, and Trebbiano giallo, right) after a fumigation with 80 ppb of O_3_ for 5 h day^-1^ (8:00–13:00 h) and 40 ppb of O_3_ for 5 h day^-1^ (13:00–18:00 h) for 28 days. Data are shown as mean ± standard deviation. The measurements were taken from untreated (open circle) and treated (closed circle) plants. For each parameter, different letters indicate significant differences (*P*≤0.05). In the boxes, results of repeated measurements ANOVA are reported. Asterisks show the significance of factors (ozone and time of day) and their interaction for: *** = *P*≤0.001, ** = *P*≤0.01, * = *P*≤0.05).

A/C_i_ response curves were measured in leaves exposed to filtered air or to O_3_ after 7, 14, 21 and 28 days of treatment (Fig 8). In both varieties, interactions between O_3_ and time were always significant, as well as the effects of both single factors. In ALE, a lower V_cmax_ value was observed starting from 14 days FBE (-20% in comparison with filtered air material), which lasted for the remaining period of fumigation (-30% at 21 days FBE) (Fig 8A). Similarly, in TRE there was a reduction in V_cmax_ already 7 days FBE (-27%). The decline reached a maximum of -42% at 21 and 28 days FBE (Fig 8B). J_max_ was also affected by O_3_ starting from 14 and 7 days FBE in ALE (-14%) and in TRE (-27%), respectively (Fig 8C and 8D), the greatest reduction occurring at 21 (-19%) and 28 (-42%) days FBE in ALE and TRE, respectively. In treated ALE, CO_2_ saturation point (CSP) levels decreased after 14 days FBE (-29%), reaching a maximum at the end of the treatment (-36%) (Fig 8E). In treated TRE, CSP dropped from 21 days FBE (-9%), with the greatest decrease at the end of the exposure (-12%) (Fig 8F). In both varieties, the CO_2_ compensation point (CCP) was similar in the control and fumigated plants until 14 days FBE. The maximum of reduction was observed at 28 days FBE (-44 and -22% in ALE and TRE, respectively) (Fig 8G and 8H).

### Dynamics of chlorophyll *a* fluorescence

**Fig 9:**
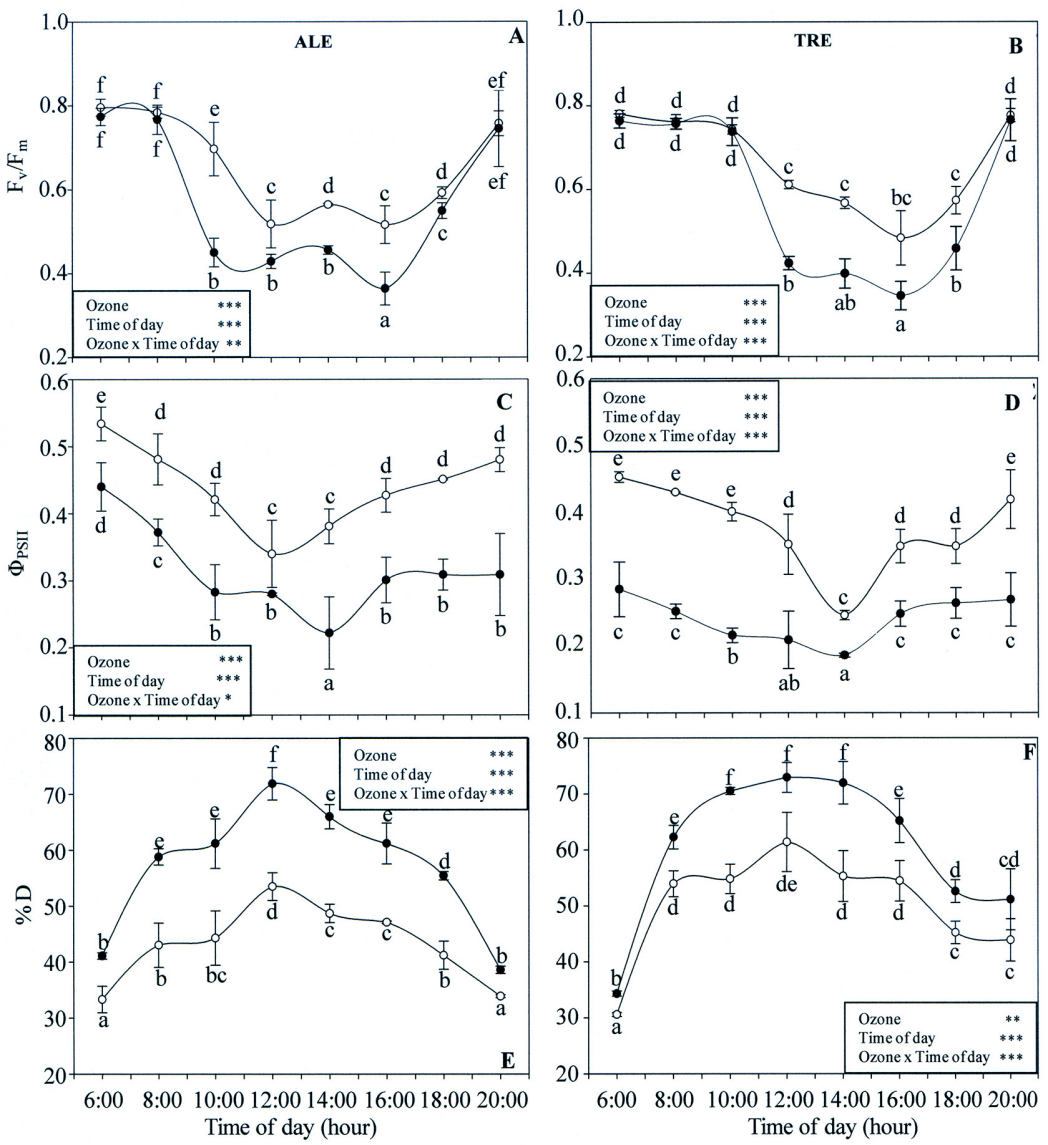
Diurnal time courses of chlorophyll *a* fluorescence parameters in *Vitis vinifera* varieties exposed or not to ozone treatment. Diurnal time courses of variable and maximal fluorescence ratio (F_v_/F_m_, A-B), actual quantum yield of PSII (Φ_PSII_, C-D) and fraction of light absorbed by PSII antenna which are thermally dissipated (%D, E-F) in *Vitis vinifera* leaves Aleatico, left, and Trebbiano giallo, right) after a fumigation with 80 ppb of O_3_ for 5 h day^-1^ (8:00–13:00 h) and 40 ppb of O_3_ for 5 h day^-1^ (13:00–18:00 h) for 28 days. Data are shown as mean ± standard deviation. The measurements were taken from untreated (open circle) and treated (closed circle) plants at the end of exposure. For each parameter, different letters indicate significant differences (*P*≤0.05). In the boxes, results of repeated measurements ANOVA are reported. Asterisks show the significance of factors (ozone and time of day) and their interaction for: *** = *P*≤0.001, ** = *P*≤0.01, * = *P*≤0.05).

Regarding the chlorophyll fluorescence parameters (Fig 9), in both varieties interactions between O_3_ and time of day were always significant; the same was true for the effects of both single factors. In ALE controls, the F_v_/F_m_ ratio was high in the early morning, then decreased with time and reached a minimum at 12:00 and 16:00 h. Afterwards, the levels gradually increased and reached approximately the initial value in late afternoon (Fig 9A). Ozone did not modify the F_v_/F_m_ profile. However the values were significantly lower between 10:00 and 18:00 h (the minimum was at 10:00 h, -35%). In TRE controls (Fig 9B), the profile was similar, with a shift towards lower levels between 12:00 and 18:00 h. The same trend was also observed following exposure, the minimum occurring at 12:00 h (-31%). In both varieties, the daily time course of Φ_PSII_ decreased in the morning (reaching a minimum at 12:00 and 14:00 h in ALE and TRE, respectively) and increased in the afternoon (Fig 9C and 9D). Under oxidative stress, Φ_PSII_ levels were significantly reduced for the whole day [maximum decrease at 14:00 (-42%) and 10:00 h (-46%) in ALE and TRE respectively]. In both varieties (Fig 9E and 9F), %D values were lower in the early morning, increasing with time and reaching a maximum at 12:00 h. Afterwards, levels gradually decreased to the initial values. Although treated plants exhibited the same profiles as the controls, they showed significantly higher %D levels with the maximum at 12:00 h (+34 and +19% in ALE and TRE, respectively).

### Leaf water status

The leaf water status metrics is reported in Table 3. In both varieties, O_3_ treatment induced a significant decrease in Ψ_PD_ values (-33% compared to the controls). Similarly, levels of Ψ_osm_ were also reduced (-23 and -6% in ALE and TRE, respectively). Ψ_2_ and RWC showed a marked decline only in ALE treated plants (-29 and -43%, respectively).

**Table 3:** Leaf water status parameters in *Vitis vinifera* varieties exposed or not to ozone treatment.

Grape variety	Treatment	Ψ_PD_ (MPa)	Ψ_2_ (MPa)	Ψ_osm_ (MPa)	RWC (%)
**Aleatico**	Control	-0.4±0.06	-1.2±0.06	-1.3±0.01	82±5.1
	Fumigated	-0.6±0.05	-1.5±0.06	-1.7±0.01	47±6.0
***P***		**	***	***	***
**Trebbiano giallo**	Control	-0.4±0.07	-1.0±0.06	-1.7±0.02	79±8.7
	Fumigated	-0.6±0.08	-1.2±0.12	-1.8±0.04	75±7.9
***P***		**	***	***	ns

Average values (± standard deviation) of leaf water status parameters measured on two *Vitis vinifera* varieties (Aleatico and Trebbiano giallo) fumigated with O_3_ (80 ppb for 5 h day^-1^, 8:00–13:00 h, + 40 ppb for 5 h day^-1^, 13:00–18:00 h) or maintained in filtered air for 28 days. Measurements referred to the end of the exposure. For each parameter, the data were analyzed by Student’s *t*-test. The significant differences are: *** = *P*≤0.001, ** = *P*≤0.01, * = *P*≤0.05, ns = *P>*0.05. Abbreviations: Ψ_PD_, predawn leaf water potential; Ψ_2_, midday leaf water potential; Ψ_osm_, osmotic water potential; RWC, relative water content. Ψ_PD_, Ψ_osm_ and RWC were measured at 6:00 h; Ψ_2_ was measured at 14:00 h.

### Osmolytes and ABA content

**Fig 10:**
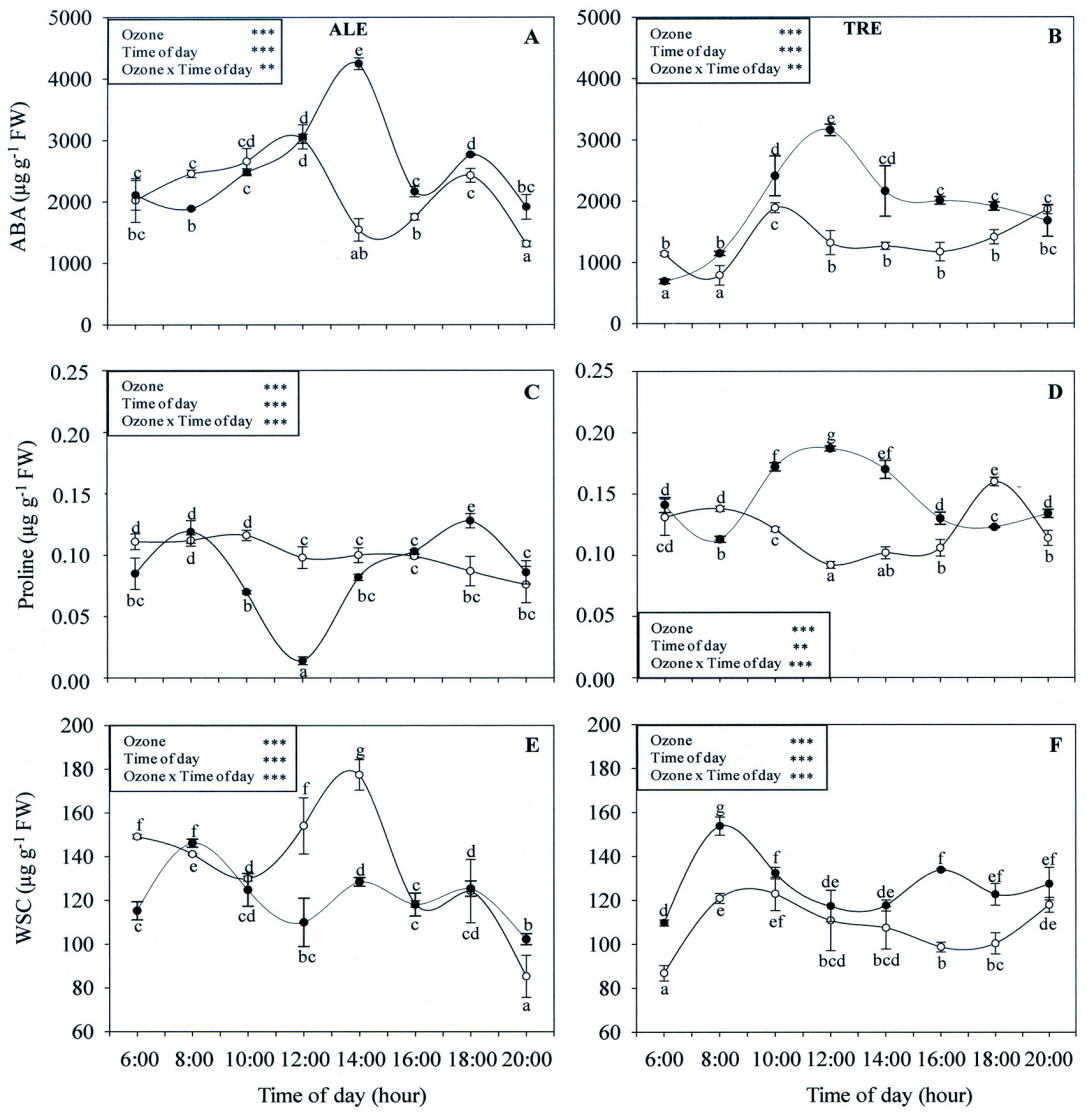
Diurnal time courses of osmolytes content in *Vitis vinifera* varieties exposed or not to ozone treatment. Diurnal time courses of abscisic acid (ABA, A-B), proline (C-D) and water soluble content (WSC, E-F) in *Vitis vinifera* leaves Aleatico, left, and Trebbiano giallo, right) after a fumigation with 80 ppb of O_3_ for 5 h day^-1^ (8:00–13:00 h) and 40 ppb of O_3_ for 5 h day^-1^ (13:00–18:00 h) for 28 days. Data are shown as mean ± standard deviation. The measurements were taken from untreated (open circle) and treated (closed circle) plants at the end of exposure. For each parameter, different letters indicate significant differences (*P*≤0.05). In the boxes, results of repeated measurements ANOVA are reported. Asterisks show the significance of factors (ozone and time of day) and their interaction for: *** = *P*≤0.001, ** = *P*≤0.01).

Diurnal variations in osmolyte content are reported in Fig 10. The two-way ANOVA test revealed that the interaction between O_3_ and time of day was significant for all parameters and for both varieties; the same was true for the effects of both factors. In ALE controls, levels of ABA showed a biphasic diurnal pattern (Fig 10A). The concentrations were low in the early morning, increasing with time and reaching a maximum at 12:00 h. A second peak was observed at 18:00 h. ABA content measurements in the morning showed only minimal differences between treated and control plants. At 14:00 h, the ABA concentration was always significantly higher in treated individuals (+175%). In TRE controls (Fig 10B), the ABA circadian profile showed a single peak at 10:00 h. Ozone increased the ABA level throughout the whole day (except for 6:00 and 20:00 h), the maximum difference being at 12:00 h (+140%).

In ALE controls, the proline levels showed similar values throughout the day (Fig 10C). Treated plants exhibited a marked drop at 12:00 h (-86%) and a rise at 18:00 h (+47%). A more fluctuating trend was evidenced in TRE controls (Fig 10D), with the lowest concentrations around midday and the highest in the afternoon. Ozone led to a marked increase in the proline level, above all around midday (+103%).

In untreated ALE (Fig 10E), the diurnal profile of the WSC content showed a sharp peak between 12:00 and 14:00 h. Ozone induced a reduction in these concentrations (-29 and -27 at 12:00 and 14:00, respectively). In TRE controls (Fig 10F), the highest WSC values were observed in the morning (between 8:00 and 10:00) and in the evening. Treatment with O_3_ significantly increased the WSC content in the early morning (+27% at 8:00 h) and in the afternoon (+35% at 16:00 h).

### Photosynthetic pigments

**Fig 11:**
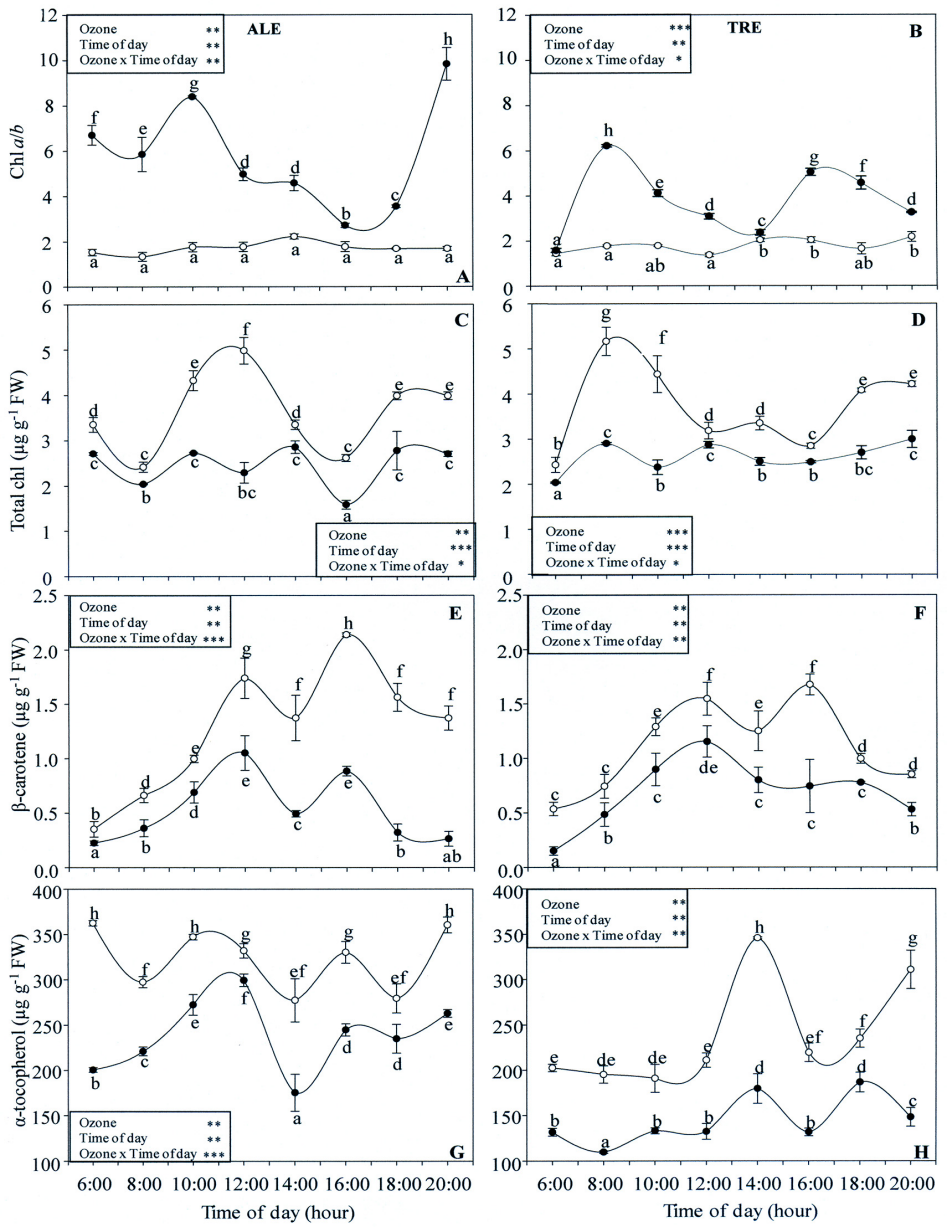
Diurnal time courses of photosynthetic pigments in *Vitis vinifera* varieties exposed or not to ozone treatment. Diurnal time courses of chlorophyll *a*/*b* ratio (chl *a*/*b*, A-B), total chlorophyll (tot chl, B-C), β-carotene (D-E) and α-tocopherol (F-G) in *Vitis vinifera* leaves (Aleatico, left, and Trebbiano giallo, right) after a fumigation with 80 ppb of O_3_ for 5 h day^-1^ (8:00–13:00 h) and 40 ppb of O_3_ for 5 h day^-1^ (13:00–18:00 h) for 28 days. Data are shown as mean ± standard deviation. The measurements were taken from untreated (open circle) and treated (closed circle) plants at the end of exposure. For each parameter, different letters indicate significant differences (*P*≤0.05). In the boxes, results of repeated measurements ANOVA are reported. Asterisks show the significance of factors (ozone and time of day) and their interaction for: *** = *P*≤0.001, ** = *P*≤0.01, * = *P*≤0.05).

In both varieties, interactions between O_3_ and the time of day, as well as the effects of both single factors were always significant (Fig 11). In ALE controls, the chlorophyll *a*/chlorophyll *b* ratio (chl *a*/*b*) showed similar values throughout the day (Fig 11A). Under O_3_ pressure, diurnal variations were expressed by a double-peak profile: the first peak appeared at 10:00 h (about 5-fold higher in comparison to control) and the second one at 20:00 h (about 6-fold). However, in treated plants the other values were also significantly higher. In both fumigated and control TRE (Fig 11B), chl *a*/*b* behaved the same as in ALE. In treated TRE, the first peak was about 4-fold (at 8:00 h) and the second about 2.5-fold (between 16:00 and 18:00 h) higher than controls. In controls of both varieties, the diurnal profile of the total chlorophyll content (tot chl) was a biphasic curve. In ALE, maximum values were observed between 10:00 and 12:00 h and again between 18:00 and 20:00 h (slightly lower) (Fig 11C). In TRE, the maximum content was at 8:00 h and between 18:00 and 20:00 h (Fig 11D). In both varieties, O_3_ induced a marked reduction in tot chl during the whole day, the maximum differences occurring at 12:00 h (-54%) and at 8:00 h (-53%) in ALE and TRE, respectively.

For β-carotene, the diurnal profile was similar in controls of both varieties (Fig 11E and 11F), and two peaks were detected at 12:00 and 16:00 h. Treated ALE maintained the same profile as the controls, but with lower values: the maximum decrease occurred at 20:00 h (-76%). In treated TRE, the profile showed a single peak: the maximum difference was at 16:00 h (-61%).

In control ALE, the α-tocopherol content fluctuated, with the highest values at 6:00, 10:00 and 20:00 h. In treated ALE, α-tocopherol levels were lower during the whole day, the maximum difference being at 6:00 h (-45%) (Fig 11G). In control TRE, the profile was biphasic, with peaks at 14:00 and 20:00 h. Ozone affected α-tocopherol content with a marked drop during the whole day, the maximum reduction occurring at 20:00 h (-52%) (Fig 11H).

**Fig 12:**
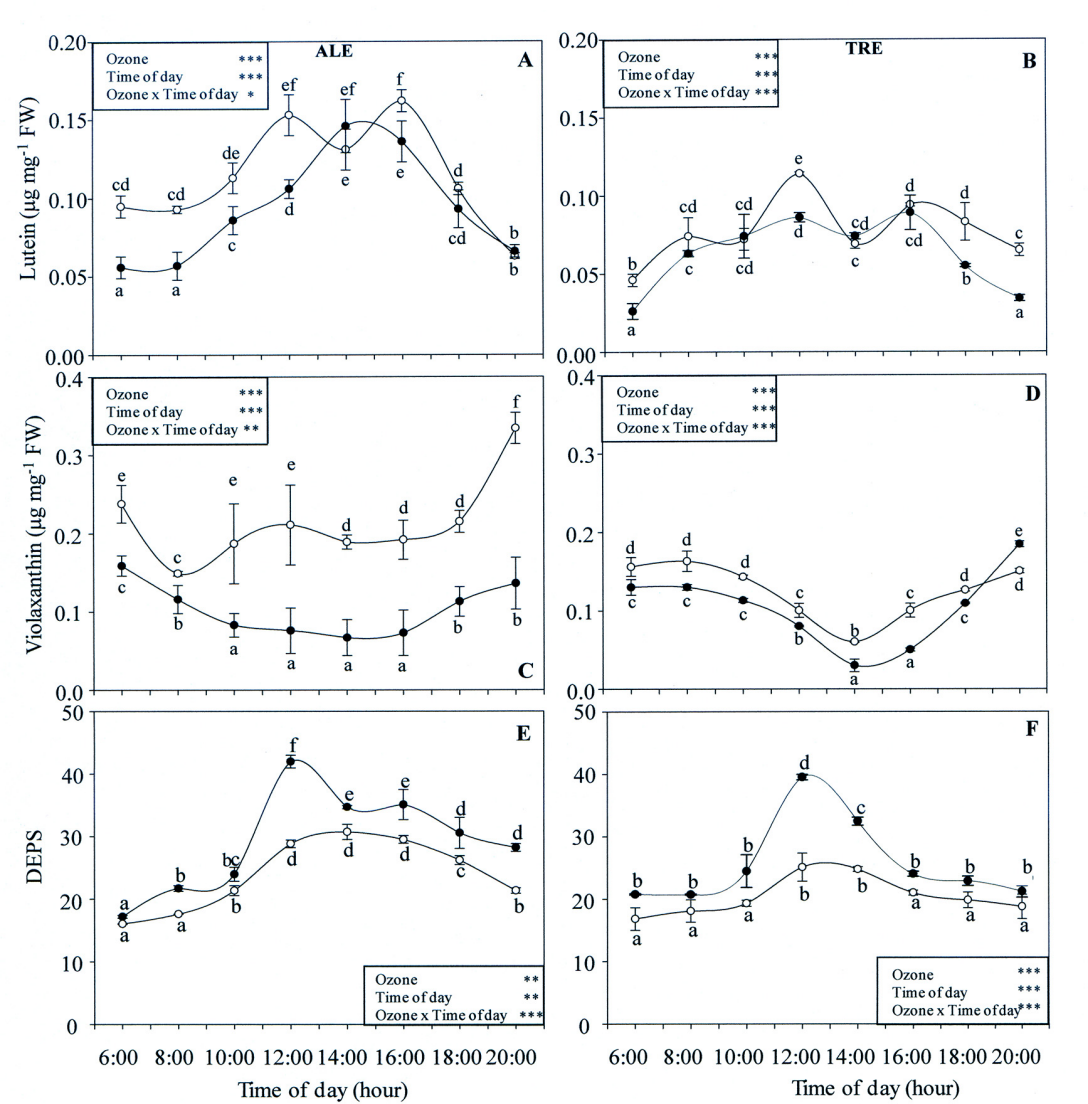
Diurnal time courses of accessory pigments in *Vitis vinifera* varieties exposed or not to ozone treatment. Diurnal time courses of lutein (A-B), violaxanthin (C-D) content and de-epoxidation index (DEPS, E-F) in *Vitis vinifera* leaves Aleatico, left, and Trebbiano giallo, right) after a fumigation with 80 ppb of O_3_ for 5 h day^-1^ (8:00–13:00 h) and 40 ppb of O_3_ for 5 h day^-1^ (13:00–18:00 h) for 28 days. Data are shown as mean ± standard deviation. The measurements were taken from untreated (open circle) and treated (closed circle) plants at the end of exposure. For each parameter, different letters indicate significant differences (*P*≤0.05). In the boxes, results of repeated measurements ANOVA are reported. Asterisks show the significance of factors (ozone and time of day) and their interaction for: *** = *P*≤0.001, ** = *P*≤0.01, * = *P*≤0.05).

The levels of the other accessory pigments are reported in Fig 12. In both varieties, interactions between O_3_ and time of day were always significant; and the same was true for the effects of single factors. In untreated ALE, the highest content of lutein was observed between 12:00 and 16:00 h. This behavior was also observed in ALE exposed to O_3_, the peak being between 14:00 and 16:00 h. The maximum reduction was at 6:00 h (-41% compared to control) (Fig 12A).

In TRE controls, the diurnal profile of lutein content was a single-peak curve with a maximum at 12:00 h. Ozone induced a decrease in the early morning, at midday and in the evening (-44, -25, -34 and -48% at 6:00, 12:00, 18:00 and 20:00 h, respectively) (Fig 12B). In control ALE, the daily time course of violaxanthin content showed a steady trend, with maximum values in the early morning and evening. The consequence of O_3_ exposure was a reduction in levels during the whole day, with a maximum decrease at 14:00 h (-65%) (Fig 12C). Also in TRE controls, violaxanthin levels were about the same during the whole day. After O_3_ exposure, the profile did not change, but lower levels occurred, the maximum difference being between 14:00 and 16:00 h (-40%) (Fig 12D).

The de-epoxidation index (DEPS) of untreated ALE showed a single-peak diurnal pattern (Fig 12E), with the maximum between 12:00 and 16:00 h. O_3_ had an evident effect on DEPS throughout the day (except at 6:00 h), with a maximum increase at 12:00 h (+46% in comparison to control). Also in TRE controls (Fig 12F), the behaviour was the same as observed in ALE, with a maximum between 12:00 and 14:00 h. Also in this case, O_3_ induced an increase in DEPS throughout the day, with a marked rise at 12:00 h (+57%).

## Discussion

Plant response mechanisms to oxidative pressure have been related to morphological characteristics, which can affect the degree of acclimation and plant ability to support detoxification. Usually, leaves that present (i) low stomata density, (ii) high values of LMA, (iii) high thickness and (iv) high values of V_cmax_ and J_max_ could be characterized by considerable longevity [39–40]. These anatomical features would seem to improve water-dissipation and leaf resistance to gas exchange [41], suggesting that TRE is characterized by a potential higher degree of acclimation to an oxidative environment than ALE.

In O_3_-exposed and symptomatic leaves, some anatomical alterations take place, which are indicative of oxidative processes associated with the apoplastic oxidative burst [42]. Particularly in O_3_-fumigated ALE, the (i) lower values of palisade thickness compared with the controls (depending on the anomaly in the size of spongy mesophyll cells), (ii) degradation of thylakoid system, and (iii) increase in plastoglobuli number and size, could be related to the premature tissue ageing induced by oxidative stress, in line with similar results observed in other species [4; 43–45].

The extent of the photosynthetic response of grapevine varieties to oxidative stress is characterized by high heterogeneity and depends on complex interactions between photoinhibitory damage, repair, photoprotection and acclimation of the photosynthetic machinery [46]. In our study, the picture provided by gas exchange measurements was characterized by a similar qualitative and quantitative response in the two varieties. Following O_3_ exposure, the assimilatory apparatus of leaves showed a midday depression of A which is usually reported under natural conditions [47]. These values strongly decreased throughout the day. This reduction took place along with a partial stomata closure and storage of CO_2_ in substomatal cavities, independently of PPFD.

Ecophysiological and cytological data indicated that stomatal and mesophyll processes may be involved in reducing A values and that there is a close co-ordination between them. In O_3_-fumigated leaves of both varieties, the inhibition of photosynthetic activity was associated to (i) impairment of stomatal function, and (ii) disorder in mesophyll CO_2_ fixation ability. This behavior has been observed by other authors [48–49]. It is not clear whether the O_3_-induced reduction in G_w_ could be the result (indirect effect via A) or the cause of decreased photosynthesis (direct effect of O_3_ on the stomata).

In both our varieties, the loss of A capacity in treated leaves was correlated with the slow-down of the dark reactions of the Calvin cycle, which, in turn, was mainly due to the loss of carboxylation efficiency. In O_3_-fumigated ALE, the marked increase in C_i_ (when Ψ_2_ is low) suggests a decrease in activity of CO_2_ assimilation mechanisms, with a decrease in carboxylation efficiency. The weekly results obtained by the A versus C_i_ curves showed a good agreement between mesophyll versus stomatal effects. In both varieties, the clear decrease in some parameters obtained by the A/C_i_ curves suggests, according to previous studies [50–51], that a reduction in the carboxylation capacity is one of the primary changes responsible for a decrease in CO_2_ uptake in O_3_-fumigated plants. In addition, the behavior of photosynthetic response curves to increasing irradiance showed that during and following the treatment, O_3_ induced an alteration in CO_2_ assimilation both at low (as indicated by the increase in LCP) and high (as confirmed by the decrease in LSP) light intensity in both varieties. In O_3_-fumigated ALE, the decrease in A_max_ was more rapid and greater, indicating that TRE may be less sensitive to oxidative stress.

A consequence of the reduction in photosynthetic capacity caused by O_3_ is the exposure of the plant to excess energy, which, if not safely dissipated, can lead to changes in the functional state of the thylakoid membranes. This can modify the characteristics of the fluorescence signals, which can be quantified in the leaves, providing data to estimate the inhibition or damage in the process of electron transfer in PSII [52].

In O_3_-treated leaves of both varieties, a reversible decline in the F_v_/F_m_ ratio was found during the day (without qualitative modifications in the circadian profile in comparison to controls), suggesting that a complete PSII repair/activation occurred when favorable meteo-climatic conditions were restored. The O_3_-induced reduction in this parameter could be due to the down-regulation of PSII activity (as indicated by the reduction in Φ_PSII_ over the entire day) rather than to photodamage to PSII.

These results would thus be consistent with the idea that there is a tendency to reduce the light energy used in photochemistry by adapting ATP and NADPH production to the reduced demand of the Calvin cycle. This conclusion is supported by similar findings described, for instance, in maple [53] and bean [54] plants exposed to O_3_. Under these conditions, photoinhibition may be avoided by decreasing the absorption of light and/or consuming the excess excitation energy through non-photochemical mechanisms [55], as confirmed by the activation of heat energy dissipation (increase of %D and DEPS). In TRE untreated leaves, the observed high %D levels could be a key factor contributing to the lower sensitivity of this variety to oxidative stress. Similar results are reported in the literature for white clover [50] and bean [54, 56] genotypes.

The general reduction during the whole day in chlorophyll content exhibited in O_3_-treated leaves of both varieties has been suggested as damage (indicated by foliar injury) and not as a photoprotective mechanism. Despite the decrease in the total chlorophyll content, the chl *a*/*b* ratio significantly increased, and β-carotene and α-tocopherol contents decreased, thus indicating a rearrangement of the pigment composition of the photosynthetic apparatus [57]. This hypothesis is also confirmed by the concomitant increase in DEPS throughout the day and may be linked to a moderate reduction in light-harvesting complex proteins (LHCPs) and with an enrichment in pigment-protein complexes characterized by a relatively high chl *a*/*b* ratio [58].

In ALE, compared to TRE, the chlorophyll antenna size and the number of functioning photosynthetic units were rapidly (the chl *a*/*b* ratio had already increased in the early morning) and markedly modified (significant drop in β-carotene and α-tocopherol content). However, the re-organisation of the photosynthetic apparatus did not preserve the PSII photochemistry of O_3_-treated ALE. Similar results have been reported in *Phaseolus vulgaris* plants exposed to non-filtered air supplied with 80 ppb of O_3_ [59].

The net α-tocopherol loss suggests that ALE is O_3_-stress sensitive: under oxidative stress conditions, α-tocopherol degradation exceeds its synthesis, so inducing lipid peroxidation and cell damage. The interdependence between α-tocopherol and oxygenated carotenoids in the protection of the photosynthetic machinery (at the structural and functional levels) has been demonstrated [60–61]. The reduction in β-carotene, lutein and α-tocopherol may be triggered by the oxidative cleavage of the carotenoids which can lead to the production of ABA.

This hypothesis is also confirmed by the concomitant decrease in the violaxanthin content observed during the whole day in both varieties. This pigment (a precursor of ABA) can be used to synthesize the hormone under oxidative stress conditions, and this metabolic shift could make violaxanthin less available as a substrate for the xanthophyll cycle reaction. ABA has long been recognized as a stress hormone and plays an important role in the physiological adaptation of plants under unfavorable environmental conditions [35, 62]. In O_3_-treated ALE, the high ABA levels monitored in the early afternoon indirectly maintain constant stomatal conductance to (i) control the transpiration, diminish (ii) water losses, and (iii) CO_2_ uptake. When favorable climatic conditions are restored, proline and WSC accumulation occurred which could be mediated by an ABA-dependent pathway [63]. This is confirmed by the stimulation of this hormone that possibly coordinates the withdrawal of water from the guard cells in favor of the subsidiary cells, and may then lead to stomatal closure.

The skin cracks on the leaf epidermis and the concomitant decline in Ψ_PD_ and Ψ_osm_ values suggest that the ABA-dependent (i) regulation of the stomata and (ii) modulation of water relations (in O_3_-treated leaves ABA accumulated prior to proline accumulation) do not prevent the water deficit (reflecting O_3_-induced injury).

In contrast, the elevated levels of ABA observed during the light-period in O_3_-treated TRE could be considered as the main controlling factor for stomatal responses to oxidative stress, according to the results we obtained by gas exchange analyses. This accumulation could regulate the stimulation of proline synthesis from glutamic acid (which has been found to be dependent on ABA concentration). This is confirmed by the significant increase in this organic solute at midday, which may function to indirectly protect PSII [64], and the concomitant rise of WSC.

Similar conclusions have been reported in plants exposed to drought [65] and salinity stress [66]. The accumulation of compatible solutes, such as proline, is one of the mechanisms used to modulate water relations [67]. In O_3_-treated TRE, the increase in ABA takes place simultaneously with that of proline. Thus, the endogenous content of ABA is probably involved in the regulation of proline metabolism resulting in partial maintenance of leaf water content (Ψ_2_ and RWC not affected).

## Conclusions

In relation to their morphological functional traits, ALE and TRE manage to acclimate to oxidative pressure induced by a long-term O_3_ exposure. At the constitutive levels of TRE, the high values of leaf area, thickness and mass per area can be correlated to the higher degree of tolerance to O_3_. At the physiological levels, both varieties are able to support and activate similar detoxification mechanisms such as xanthophyll cycle-dependent thermal energy dissipation and photorespiration. Slight genotypic differences were observed only in terms of constitutive photosynthesis capacity per surface unit (as confirmed by the high values of V_cmax_ and J_max_ in TRE). These differences do not have biological relevance and do not contribute to explain the behavior of variety in response to O_3_. In addition, ALE reacts with morphological changes, i.e. irregular LT, presence of skin cracks on the epidermis, reduction in cell contact in the palisade layer, degradation of the thylakoid system. These changes are detrimental for photosynthetic function due to non stomatal limitations, and for leaf water status, as confirmed by the decrease in RWC and Ψ_2_ values.

We can conclude that firstly the daily photosynthetic performance of grapevine leaves was affected by realistic exposure to O_3_. The picture provided by gas exchange and chlorophyll *a* fluorescence measurements was characterized by a similar qualitative response in the two varieties. Secondly, the genotypic variability of *V*. *vinifera* and the functional leaf traits regulated the acclimatory response to oxidative stress and the degree of tolerance to O_3_. Finally, similar photoprotective mechanisms were activated in the two varieties (xanthophyll cycle-dependent thermal energy dissipation, photorespiration and stomatal closure) although their extent is characterized by high heterogeneity. In comparison to ALE, TRE was less sensitive to oxidative stress induced by O_3_ because of the morphological functional traits that regulate the degree of tolerance to abiotic stress (in terms of visible injury) and the acclimatory response.
